# Preventive practices toward sexually transmitted infections and their determinants among young people in Ethiopia: A protocol for systematic review and meta-analysis

**DOI:** 10.1371/journal.pone.0262982

**Published:** 2022-02-03

**Authors:** Etsay Woldu Anbesu, Setognal Birara Aychiluhm, Mussie Alemayehu

**Affiliations:** 1 Department of Public Health, College of Medicine and Health Sciences, Samara University, Samara, Ethiopia; 2 School of Public health, College of Health Science, Mekelle University, Mek’ele, Tigray, Ethiopia; Babol University of Medical Science, ISLAMIC REPUBLIC OF IRAN

## Abstract

**Background:**

Globally, the estimated annual number of new cases of curable sexually transmitted infections occurring among young people aged 15–24 years is approximately 178.5 million. There are fragmented and inconsistent findings on preventive practices for sexually transmitted infections. Thus, this systematic review and meta-analysis protocol aimed to estimate the pooled prevalence of preventive practices of sexually transmitted infections and identify its determinants among young people in Ethiopia.

**Methods:**

The Preferred Reporting Items for Systematic Review and Meta-analyses (PRISMA) will be used to develop the review protocol. Online databases such as PubMed, CINAHL, Scopus, Google, and Google Scholar will be used to search published and unpublished studies. The Joanna Briggs Institute Meta-Analysis of Statistics Assessment and Review Instrument will be used to assess the quality of the study. Statistical heterogeneity will be checked using the Cochran Q test and I^2^ statistics. Subgroup analysis and meta-regression will be performed to identify the sources of heterogeneity. The statistical analysis will be performed using STATA version 14 software. A random-effects model will be performed to estimate the pooled prevalence and identify determinants of preventive practices of sexually transmitted infections.

**Discussion:**

Young people have a high unmet need for sexual and reproductive health services and poor preventive practices toward sexually transmitted infections. Although there are studies on preventive practices for sexually transmitted infections, there is no study finding on the pooled prevalence of preventive practices for sexually transmitted infections and its determinants among young people in Ethiopia. Thus, this systematic review and meta-analysis protocol will help to develop appropriate strategies.

## Introduction

Sexually transmitted infections (STIs) are diseases such as gonorrhea, syphilis, chancroids, lymphogranuloma venerum, and more than 30 different bacteria, viruses, and parasites. STIs can be curable and incurable. The curable STIs include gonorrhea, syphilis, trichomonas, and chlamydia. The incurable STIs include herpes simplex virus, hepatitis B, human immunodeficiency virus (HIV), and human papilloma virus (HPV). STIs are transmitted through sexual contact, such as vaginal, anal, and oral sex. It can also spread through nonsexual means via blood or blood products and mother to fetus during pregnancy, childbirth, and breastfeeding. The common symptoms or syndromes of STIs include urethral discharge, vaginal discharge, genital ulcer, lower abdominal pain, inguinal bubo, neonatal conjunctivitis, and scrotal swelling [1–4].

Globally, the estimated annual number of new cases of curable STIs that occur among people aged 15–49 years is 357 million, and approximately half of them are between 15–24 years [5, 6]. Every day, more than 1 million STIs are acquired [1]. STIs are health threats to adolescents and young people in developed and developing countries [4, 7–11]. In Ethiopia, although there is a lack of surveillance data, the self-reported prevalence of STIs among female and male youth aged 15–24 years was 3% and 1%, respectively [12]. STIs cause serious consequences, including an increased risk of HIV infection, stillbirth, neonatal death, low birth weight, sepsis, pneumonia, and neonatal conjunctivitis or blindness [1].

The age of young people by itself is a risk factor for many factors. It is a critical developmental period where youth begin to know and explain their sexual values and behaviors. They are at high risk for unsafe sexual behaviors, including STIs, unplanned pregnancy, abortion, low school performance, psychosocial problems, and economic crises [1, 13]. Moreover, rapid reproductive maturity among young people could lead to early sexual initiation and unsafe sex with the reluctance to use contraceptive methods [14–17].

In addition, factors such as multiple sexual partners, engaging in risky sexual behavior (RSB), sex without condoms, sex with older partners, consumption of alcohol and illicit drugs, cultural, religious, peer pressure, watching pornography, being single, nondisclosure of HIV status, and conflicts between couples and families affect young people’s sexual behavior [4, 8, 10, 18–24].

In Ethiopia, although STIs remain one of the sustainable development goal (SDG) agendas [25] and the development of national guidelines for the management of STIs using a syndromic approach [26], there is a lack of attention and surveillance data on preventive practice toward STIs in young people [27]. Moreover, although studies have been conducted in different parts of the country on the preventive practices of STIs among young people, there are inconsistent findings on prevalence and its determinants. Thus, this systematic review and meta-analysis protocol aimed to estimate the pooled prevalence of preventive practices of STIs and identify its determinants among young people in Ethiopia.

## Research question

- What is the pooled prevalence of preventive practices toward STIs among young people in Ethiopia?
- What are the determinants of preventive practices toward STIs among young people in Ethiopia?

## Objectives

- To determine the pooled prevalence of preventive practices toward STIs among young people in Ethiopia
- To identify the determinants of preventive practices toward STIs among young people in Ethiopia

## Methods

### Review protocol development

The Preferred Reporting Items for Systematic Review and Meta-analyses (PRISMA) will be used to develop the review protocol [28], and the PRISMA-P 2015 checklist will be used to report the protocol procedures [29] (S1 File).

### PECO search guide

#### Population

Young people (10–24 years old) [30].

#### Exposure

Exposure is a determinant that increases or decreases the likelihood of preventive practices toward sexually transmitted infections.

#### Comparison

The reference group for each determinant in each study will be the comparison variable. It may include good knowledge versus poor, positive attitude versus negative, education versus no education, access to information versus no accesses, consistent use of condoms versus not.

#### Outcome

The outcome variable will be the pooled prevalence of preventive practices of STIs. Studies with the primary objective of determining the prevalence of preventive practices of STIs and their determinants among young people in Ethiopia will be considered.

### Data source and search strategies

Online databases such as PubMed, CINAHL, Scopus, Google, and Google Scholar will be used to search published and unpublished studies. As searching Google and Google Scholar leads to numerous studies, a limited number of studies will be screened from Google and Google Scholar using the phrase “Preventive practices toward Sexually Transmitted Infection in Ethiopia”. The two authors (EW and SB) will retrieve the studies. In addition, across-reference search will be performed to add other related studies from the final included studies that may be missed during the database search. The search terms are indicated in S2 File. The search string will be adapted based on the specific requirements of the database to identify relevant studies. Retrieve studies will be exported to Endnote version 8 reference Manager software [31].

### Eligibility criteria

All observational studies (cross-sectional, case–control, and cohort) will be included in the systematic review and meta-analysis. Studies that reported the prevalence of preventive practices of STIs and its determinants among young people in Ethiopia will be included. Moreover, studies that reported only the prevalence of preventive practices of STIs or at least measured associations between determinant variables and the preventive practice of STIs will be considered. Institutional and community-based studies will be included. We will exclude studies that only address the qualitative approach. For both quantitative and qualitative data, only the quantitative data will be considered. We will not make restrictions on the date of publication. Studies published other than those in the English language, expert opinions, conferences, and case reports will be excluded.

### Selection of studies

The two authors (EW and SB) will independently screen the studies based on the titles and abstracts. Duplicate, irrelevant title, and abstract studies will be removed from the citation manager. The quality of full text studies will be evaluated, and studies that are not eligible will be removed. We will discuss with the third author (MA) to resolve any disagreement among reviewers during the review process. The selection process flow diagram will be presented using the PRISMA chart (S3 File).

### Quality assessment

To assess the quality and validity of the studies, the Joanna Briggs Institute Meta-Analysis of Statistics Assessment and Review Instrument (JBI-MAStARI) will be used [32]. The quality assessment will focus on clear inclusion criteria, study subjects and setting, standard measurement criteria, exposure and outcome measurements, and appropriate statistical analysis (S4 File). The quality of the studies will be assessed independently by the two authors (EW and SB). Studies 50% and above of the quality scale will be considered for the final systematic review and meta-analysis. During the quality review of the studies, any disagreement among reviewers will be resolved with the third author (MA).

### Data extraction

A data extraction template form on Microsoft Excel (2016) will be used. Before the beginning of the actual data extraction, we will pilot the Microsoft Excel data extraction form. The first author’s name, publication year, study area, study design, sample size, associated factors, odds ratio, and prevalence of the studies will be included in the data extraction template. In addition, we will calculate the logarithm and standard error (SE) of the prevalence and odds ratio. The two authors (EW and SB) will extract the data independently. Discussion will be made for any difference with a third author (MA). We will contact the corresponding author of the studies in case of missing data or incomplete reports.

### Data synthesis and statistical analysis

Narrative synthesis of data will be done for the included studies. Summary tables and graphs will be performed to describe the characteristics of the included studies. Random-effects model [33] will be performed to estimate the overall pooled prevalence of preventive practices ofSTIs and identify its determinants among youths in Ethiopia. A 95% CI will be used to declare the statistical significance. Statistical heterogeneity will be checked using the Cochran Q test [34] and I^2^ statistics [35]. I^2^ values represent 25% low, 50% moderate, and 75% substantial heterogeneity. Subgroup nalysis and meta-regression will be done toidentify the sources of heterogeneity. Sensitivity analysis will be performed to assess the effect ofstudies on the overall estimation. The presence of publication bias will be checked usingfunnel plot [36], Egger’s, and Beggar’s test [37].

## Discussion

This systematic review and meta-analysis protocol aimed to estimate the pooled prevalence of preventive practices of STIs and identify its determinants among young people in Ethiopia. Although young people are sexually active, they have an unmet need for sexual and reproductive health services. The common barriers in low- and middle-income countries include lack of behavioral change and accessibility of services [38–40]. Despite different interventions implemented to enhance the preventive practice of STIs among young people, the problem is still challenging in low-income countries, including Ethiopia [12, 26].

To the best of our knowledge, there is no study finding on the pooled prevalence of preventive practices of STIs and its determinants among young people in Ethiopia. Thus, this systematic review and meta-analysis protocol will help policymakers develop appropriate interventions on preventive practices of STIs in Ethiopia. This study protocol may have limited heterogeneity between studies. Only observational study designs published in the English language will be included.

## Supporting information

S1 File PRISMA-P 2015 checklist. (DOCX)

S2 File Draft of search strategy to be used using PubMed electronic database. (DOCX)

S3 File Diagrammatic presentation of the studies selection process for systematic review. (DOCX)

S4 File JBI critical appraisals for observational studies as shown in the link below https://jbi.global/critical-appraisal-tools. (DOCX)

## References

[1] WHO, *Sexually transmitted infections (STIs)*. Fact Sheets, 2019.

[2] Lazarus J.V., et al., Systematic review of interventions to prevent the spread of sexually transmitted infections, including HIV, among young people in Europe. Croatian medical journal, 2010. 51(1): p. 74–84. doi: 10.3325/cmj.2010.51.74 20162748 PMC2829175

[3] Organization, W.H., *Report on global sexually transmitted infection surveillance 2018*. 2018.

[4] Samkange-Zeeb F.N., Spallek L., and Zeeb H., Awareness and knowledge of sexually transmitted diseases (STDs) among school-going adolescents in Europe: a systematic review of published literature. BMC public health, 2011. 11(1): p. 1–12. doi: 10.1186/1471-2458-11-727 21943100 PMC3189891

[5] Organization, W.H., *Strengthening the health sector response to care*, *support*, *treatment and prevention for young people living with HIV*: *WHO/UNICEF consultation*, *13–17 November 2006 Blantyre*, *Malawi*. 2008, World Health Organization.

[6] Organization, W.H., *Global health sector strategy on sexually transmitted infections 2016–2021*: *toward ending STIs*. 2016, World Health Organization.

[7] Chanakira E., et al., Factors perceived to influence risky sexual behaviours among university students in the United Kingdom: a qualitative telephone interview study. BMC public health, 2014. 14(1): p. 1–7. doi: 10.1186/1471-2458-14-1055 25300195 PMC4203964

[8] Chen M., et al., Comparison of sexual knowledge, attitude, and behavior between female Chinese college students from urban areas and rural areas: a hidden challenge for HIV/AIDS control in China. BioMed research international, 2016. 2016. 10.1155/2016/8175921 PMC5215479 28101513

[9] Goundry A.L.R., Finlay E.R., and Llewellyn C.D., Talking about links between sexually transmitted infections and infertility with college and university students from SE England, UK: a qualitative study. Reproductive health, 2013. 10(1): p. 1–7. doi: 10.1186/1742-4755-10-47 24020982 PMC3847203

[10] Hong Z., et al., Contraceptive knowledge, attitudes and behavior about sexuality among college students in Beijing, China. Chinese medical journal, 2012. 125(6): p. 1153–1157. 22613546

[11] Matkins P.P., Sexually transmitted infections in adolescents. North Carolina medical journal, 2013. 74(1): p. 48–52. 23530381

[12] FMOH, *FMOH Central Statistical Agency*, *Ethiopia demographic and health survey*, *Addis Ababa*. 2016.

[13] Netsanet F. and Abebe M., Risky sexual behaviors and associated factors among male and female students in Jimma Zone preparatory schools, South West Ethiopia: comparative study. Ethiopian Journal of Health Sciences, 2014. 24(1): p. 59–68. doi: 10.4314/ejhs.v24i1.8 24591800 PMC3929929

[14] Derese A., Seme A., and Misganaw C., Assessment of substance use and risky sexual behaviour among Haramaya University Students, Ethiopia. Science Journal of Public Health, 2014. 2(2): p. 102–110.

[15] Fetene N. and Mekonnen W., The prevalence of risky sexual behaviors among youth center reproductive health clinics users and non-users in Addis Ababa, Ethiopia: a comparative cross-sectional study. PloS one, 2018. 13(6): p. e0198657. doi: 10.1371/journal.pone.0198657 29879164 PMC5991709

[16] Morris J.L. and Rushwan H., Adolescent sexual and reproductive health: The global challenges. International Journal of Gynecology & Obstetrics, 2015. 131: p. S40–S42. doi: 10.1016/j.ijgo.2015.02.006 26433504

[17] Temesgen G. and Markos Y., Assessment of substance use and risky sexual behaviour among public college students in Bonga town, Southwest Ethiopia. Am J Biomed Life Sci, 2015. 3(5): p. 91–97.

[18] Bakhoum A.Y., et al., Assessment of knowledge, attitude, and practice of risky sexual behavior leading to HIV and sexually transmitted infections among Egyptian substance abusers: a cross-sectional study. Advances in Public Health, 2014. 2014. doi: 10.1155/2014/787282 25705719 PMC4332847

[19] Gebremedhin A.T., et al., Khat chewing and risky sexual behavior in Sub-Saharan Africa: a systematic review protocol. JBI Evidence Synthesis, 2013. 11(12): p. 59–67.

[20] Girmay A. and Mariye T., Risky sexual behavior practice and associated factors among secondary and preparatory school students of Aksum town, northern Ethiopia, 2018. BMC research notes, 2019. 12(1): p. 1–7. doi: 10.1186/s13104-018-4038-6 31655614 PMC6815056

[21] Melchiorre M.G., et al., Social support, socio-economic status, health and abuse among older people in seven European countries. PloS one, 2013. 8(1): p. e54856. doi: 10.1371/journal.pone.0054856 23382989 PMC3559777

[22] Mersha A., et al., Risky sexual behaviors and associated factors among preparatory school students in Arba Minch town, Southern Ethiopia. Journal of Public Health and Epidemiology, 2018. 10(12): p. 429–442.

[23] Negeri E.L., Assessment of risky sexual behaviors and risk perception among youths in Western Ethiopia: the influences of family and peers: a comparative cross-sectional study. BMC Public Health, 2014. 14(1): p. 1–12. 24690489 10.1186/1471-2458-14-301 PMC4009035

[24] Uchudi, J., M. Magadi, and M. Mostazir, *A multilevel analysis of the determinants of high risk sexual behavior (multiple sexual partners) in sub-Saharan Africa*. Social Research Methodology Centre Working Paper: Africa. London, UK: Department of Sociology, City University, 2010.

[25] Nino, F.S., *Sustainable Development Goals—United Nations*. United Nations Sustainable Development, 2015.

[26] FMOH, *National guidelines for the management of sexually transmitted infections using syndromic approach* 2015.

[27] Alfvén T., et al., Global AIDS reporting-2001 to 2015: lessons for monitoring the sustainable development goals. AIDS and Behavior, 2017. 21(1): p. 5–14. doi: 10.1007/s10461-016-1662-9 28124296 PMC5515967

[28] Moher D., et al., Preferred reporting items for systematic reviews and meta-analyses: the PRISMA statement. PLoS medicine, 2009. 6(7): p. e1000097. doi: 10.1371/journal.pmed.1000097 19621072 PMC2707599

[29] Shamseer L., et al., Preferred reporting items for systematic review and meta-analysis protocols (PRISMA-P) 2015: elaboration and explanation. Bmj, 2015. 349. doi: 10.1136/bmj.g7647 25555855

[30] UNDESA, *Definition of Youth*. *United Nations Department of Economic and Social Affairs*. 2013.

[31] Bramer W. and Bain P., Updating search strategies for systematic reviews using EndNote. Journal of the Medical Library Association: JMLA, 2017. 105(3): p. 285. doi: 10.5195/jmla.2017.183 28670219 PMC5490709

[32] Munn Z., Tufanaru C., and Aromataris E., JBI’s systematic reviews: data extraction and synthesis. AJN The American Journal of Nursing, 2014. 114(7): p. 49–54. doi: 10.1097/01.NAJ.0000451683.66447.89 25742352

[33] Berkey C.S., et al., A random‐effects regression model for meta‐analysis. Statistics in medicine, 1995. 14(4): p. 395–411. doi: 10.1002/sim.4780140406 7746979

[34] Cooper, H., L.V. Hedges, and J.C. Valentine, *The handbook of research synthesis and meta-analysis*. 2019: Russell Sage Foundation.

[35] Higgins J.P. and Thompson S.G., Quantifying heterogeneity in a meta‐analysis. Statistics in medicine, 2002. 21(11): p. 1539–1558. doi: 10.1002/sim.1186 12111919

[36] Liu J.L., The role of the funnel plot in detecting publication and related biases in meta-analysis. Evidence-based dentistry, 2011. 12(4): p. 121–122. doi: 10.1038/sj.ebd.6400831 22193659

[37] Egger M., et al., Bias in meta-analysis detected by a simple, graphical test. Bmj, 1997. 315(7109): p. 629–634. doi: 10.1136/bmj.315.7109.629 9310563 PMC2127453

[38] Menna T., Ali A., and Worku A., Effects of peer education intervention on HIV/AIDS related sexual behaviors of secondary school students in Addis Ababa, Ethiopia: a quasi-experimental study. Reproductive health, 2015. 12(1): p. 1–8. 26346980 10.1186/s12978-015-0077-9 PMC4562206

[39] Newton-Levinson A., Leichliter J.S., and Chandra-Mouli V., Sexually transmitted infection services for adolescents and youth in low-and middle-income countries: perceived and experienced barriers to accessing care. Journal of Adolescent Health, 2016. 59(1): p. 7–16. doi: 10.1016/j.jadohealth.2016.03.014 27338664 PMC5289742

[40] Seangpraw K., et al., THE EFFECT OF SEX EDUCATION AND LIFE SKILLS FOR PREVENTIVE SEXUAL RISK BEHAVIOURS AMONG UNIVERSITY OF STUDENTS THAILAND. Journal of Ayub Medical College Abbottabad, 2017. 29(4): p. 540–546. 29330973

