# Supplementary material for: Preventive practices toward sexually transmitted infections and their determinants among young people in Ethiopia: A protocol for systematic review and meta-analysis
Source: PLoS One. 2022 Feb 3;17(2):e0262982. doi: 10.1371/journal.pone.0262982 (PMC8812866; doi:10.1371/journal.pone.0262982)
Supplement: S2 File — (DOCX) [file pone.0262982.s002.docx]

**Additional file 2: Draft of search strategy to be used using PubMed electronic database**

| **Search terms** | **Search terms** | **Results** |  |
| --- | --- | --- | --- |
| Term #1 | (((((((((((((Condom*[Title/Abstract] AND ((fha[Filter]) AND (journalarticle[Filter] OR observationalstudy[Filter]) AND (fft[Filter]) AND (english[Filter]))) OR ("Sexual Abstinence"[Title/Abstract] AND ((fha[Filter]) AND (journalarticle[Filter] OR observationalstudy[Filter]) AND (fft[Filter]) AND (english[Filter])))) OR (utilization*[Title/Abstract] AND ((fha[Filter]) AND (journalarticle[Filter] OR observationalstudy[Filter]) AND (fft[Filter]) AND (english[Filter])))) OR ("Unsafe Sex"[Title/Abstract] AND ((fha[Filter]) AND (journalarticle[Filter] OR observationalstudy[Filter]) AND (fft[Filter]) AND (english[Filter])))) OR ("Sexual Partners"[Title/Abstract] AND ((fha[Filter]) AND (journalarticle[Filter] OR observationalstudy[Filter]) AND (fft[Filter]) AND (english[Filter])))) OR (Practice*[Title/Abstract] AND ((fha[Filter]) AND (journalarticle[Filter] OR observationalstudy[Filter]) AND (fft[Filter]) AND (english[Filter])))) OR (prevention*[Title/Abstract] AND ((fha[Filter]) AND (journalarticle[Filter] OR observationalstudy[Filter]) AND (fft[Filter]) AND (english[Filter])))) OR (behaviors*[Title/Abstract] AND ((fha[Filter]) AND (journalarticle[Filter] OR observationalstudy[Filter]) AND (fft[Filter]) AND (english[Filter])))) OR (Prevalence*[Title/Abstract] AND ((fha[Filter]) AND (journalarticle[Filter] OR observationalstudy[Filter]) AND (fft[Filter]) AND (english[Filter])))) OR (Prevalence*[Title/Abstract] AND ((fha[Filter]) AND (journalarticle[Filter] OR observationalstudy[Filter]) AND (fft[Filter]) AND (english[Filter])))) OR ("Human papilloma virus"[Title/Abstract] AND ((fha[Filter]) AND (journalarticle[Filter] OR observationalstudy[Filter]) AND (fft[Filter]) AND (english[Filter])))) OR ("HIV Infections"[Title/Abstract] AND ((fha[Filter]) AND (journalarticle[Filter] OR observationalstudy[Filter]) AND (fft[Filter]) AND (english[Filter])))) OR ("Gonorrhea Infection"[Title/Abstract] AND ((fha[Filter]) AND (journalarticle[Filter] OR observationalstudy[Filter]) AND (fft[Filter]) AND (english[Filter])))) OR ("Sexually Transmitted infections"[Title/Abstract] AND ((fha[Filter]) AND (journalarticle[Filter] OR observationalstudy[Filter]) AND (fft[Filter]) AND (english[Filter]))) |  |  |
| Term #2 | (((((unmarried youths*[Title/Abstract] AND ((fha[Filter]) AND (journalarticle[Filter] OR observationalstudy[Filter]) AND (fft[Filter]) AND (english[Filter]))) OR (Young Adult*[Title/Abstract] AND ((fha[Filter]) AND (journalarticle[Filter] OR observationalstudy[Filter]) AND (fft[Filter]) AND (english[Filter])))) OR (Male[Title/Abstract] AND ((fha[Filter]) AND (journalarticle[Filter] OR observationalstudy[Filter]) AND (fft[Filter]) AND (english[Filter])))) OR (Female[Title/Abstract] AND ((fha[Filter]) AND (journalarticle[Filter] OR observationalstudy[Filter]) AND (fft[Filter]) AND (english[Filter])))) OR (Adolescence*[Title/Abstract] AND ((fha[Filter]) AND (journalarticle[Filter] OR observationalstudy[Filter]) AND (fft[Filter]) AND (english[Filter])))) OR (Humans[Title/Abstract] AND ((fha[Filter]) AND (journalarticle[Filter] OR observationalstudy[Filter]) AND (fft[Filter]) AND (english[Filter]))) |  |  |
| Term #3 | ((((((((((("Unsafe Sexual Practice"[Title/Abstract] AND ((fha[Filter]) AND (journalarticle[Filter] OR observationalstudy[Filter]) AND (fft[Filter]) AND (english[Filter]))) OR ("Premarital Sex"[Title/Abstract] AND ((fha[Filter]) AND (journalarticle[Filter] OR observationalstudy[Filter]) AND (fft[Filter]) AND (english[Filter])))) OR ("Multiple Sexual Partners"[Title/Abstract] AND ((fha[Filter]) AND (journalarticle[Filter] OR observationalstudy[Filter]) AND (fft[Filter]) AND (english[Filter])))) OR (education*[Title/Abstract] AND ((fha[Filter]) AND (journalarticle[Filter] OR observationalstudy[Filter]) AND (fft[Filter]) AND (english[Filter])))) OR (Parents*[Title/Abstract] AND ((fha[Filter]) AND (journalarticle[Filter] OR observationalstudy[Filter]) AND (fft[Filter]) AND (english[Filter])))) OR ("Family Characteristics"[Title/Abstract] AND ((fha[Filter]) AND (journalarticle[Filter] OR observationalstudy[Filter]) AND (fft[Filter]) AND (english[Filter])))) OR ("oral sex"[Title/Abstract] AND ((fha[Filter]) AND (journalarticle[Filter] OR observationalstudy[Filter]) AND (fft[Filter]) AND (english[Filter])))) OR ("female sex workers"[Title/Abstract] AND ((fha[Filter]) AND (journalarticle[Filter] OR observationalstudy[Filter]) AND (fft[Filter]) AND (english[Filter])))) OR ("risk perception"[Title/Abstract] AND ((fha[Filter]) AND (journalarticle[Filter] OR observationalstudy[Filter]) AND (fft[Filter]) AND (english[Filter])))) OR (Attitudes*[Title/Abstract] AND ((fha[Filter]) AND (journalarticle[Filter] OR observationalstudy[Filter]) AND (fft[Filter]) AND (english[Filter])))) OR ("Health Knowledge"[Title/Abstract] AND ((fha[Filter]) AND (journalarticle[Filter] OR observationalstudy[Filter]) AND (fft[Filter]) AND (english[Filter])))) OR (Factors*[Title/Abstract] AND ((fha[Filter]) AND (journalarticle[Filter] OR observationalstudy[Filter]) AND (fft[Filter]) AND (english[Filter]))) |  |  |
| Term#4 | ((urban*[Title/Abstract] AND ((fha[Filter]) AND (journalarticle[Filter] OR observationalstudy[Filter]) AND (fft[Filter]) AND (english[Filter]))) OR (Rural*[Title/Abstract] AND ((fha[Filter]) AND (journalarticle[Filter] OR observationalstudy[Filter]) AND (fft[Filter]) AND (english[Filter])))) OR (Ethiopia*[Title/Abstract] AND ((fha[Filter]) AND (journalarticle[Filter] OR observationalstudy[Filter]) AND (fft[Filter]) AND (english[Filter]))) |  |  |
| Term #5 | #1 AND #2 AND #3 AND #4=  (((((urban*[Title/Abstract] AND ((fha[Filter]) AND (journalarticle[Filter] OR observationalstudy[Filter]) AND (fft[Filter]) AND (english[Filter]))) OR (Rural*[Title/Abstract] AND ((fha[Filter]) AND (journalarticle[Filter] OR observationalstudy[Filter]) AND (fft[Filter]) AND (english[Filter])))) OR (Ethiopia*[Title/Abstract] AND ((fha[Filter]) AND (journalarticle[Filter] OR observationalstudy[Filter]) AND (fft[Filter]) AND (english[Filter]))) AND ((fha[Filter]) AND (journalarticle[Filter] OR observationalstudy[Filter]) AND (fft[Filter]) AND (english[Filter]))) AND (((((((((((("Unsafe Sexual Practice"[Title/Abstract] AND ((fha[Filter]) AND (journalarticle[Filter] OR observationalstudy[Filter]) AND (fft[Filter]) AND (english[Filter]))) OR ("Premarital Sex"[Title/Abstract] AND ((fha[Filter]) AND (journalarticle[Filter] OR observationalstudy[Filter]) AND (fft[Filter]) AND (english[Filter])))) OR ("Multiple Sexual Partners"[Title/Abstract] AND ((fha[Filter]) AND (journalarticle[Filter] OR observationalstudy[Filter]) AND (fft[Filter]) AND (english[Filter])))) OR (education*[Title/Abstract] AND ((fha[Filter]) AND (journalarticle[Filter] OR observationalstudy[Filter]) AND (fft[Filter]) AND (english[Filter])))) OR (Parents*[Title/Abstract] AND ((fha[Filter]) AND (journalarticle[Filter] OR observationalstudy[Filter]) AND (fft[Filter]) AND (english[Filter])))) OR ("Family Characteristics"[Title/Abstract] AND ((fha[Filter]) AND (journalarticle[Filter] OR observationalstudy[Filter]) AND (fft[Filter]) AND (english[Filter])))) OR ("oral sex"[Title/Abstract] AND ((fha[Filter]) AND (journalarticle[Filter] OR observationalstudy[Filter]) AND (fft[Filter]) AND (english[Filter])))) OR ("female sex workers"[Title/Abstract] AND ((fha[Filter]) AND (journalarticle[Filter] OR observationalstudy[Filter]) AND (fft[Filter]) AND (english[Filter])))) OR ("risk perception"[Title/Abstract] AND ((fha[Filter]) AND (journalarticle[Filter] OR observationalstudy[Filter]) AND (fft[Filter]) AND (english[Filter])))) OR (Attitudes*[Title/Abstract] AND ((fha[Filter]) AND (journalarticle[Filter] OR observationalstudy[Filter]) AND (fft[Filter]) AND (english[Filter])))) OR ("Health Knowledge"[Title/Abstract] AND ((fha[Filter]) AND (journalarticle[Filter] OR observationalstudy[Filter]) AND (fft[Filter]) AND (english[Filter])))) OR (Factors*[Title/Abstract] AND ((fha[Filter]) AND (journalarticle[Filter] OR observationalstudy[Filter]) AND (fft[Filter]) AND (english[Filter]))) AND ((fha[Filter]) AND (journalarticle[Filter] OR observationalstudy[Filter]) AND (fft[Filter]) AND (english[Filter])))) AND ((((((unmarried youths*[Title/Abstract] AND ((fha[Filter]) AND (journalarticle[Filter] OR observationalstudy[Filter]) AND (fft[Filter]) AND (english[Filter]))) OR (Young Adult*[Title/Abstract] AND ((fha[Filter]) AND (journalarticle[Filter] OR observationalstudy[Filter]) AND (fft[Filter]) AND (english[Filter])))) OR (Male[Title/Abstract] AND ((fha[Filter]) AND (journalarticle[Filter] OR observationalstudy[Filter]) AND (fft[Filter]) AND (english[Filter])))) OR (Female[Title/Abstract] AND ((fha[Filter]) AND (journalarticle[Filter] OR observationalstudy[Filter]) AND (fft[Filter]) AND (english[Filter])))) OR (Adolescence*[Title/Abstract] AND ((fha[Filter]) AND (journalarticle[Filter] OR observationalstudy[Filter]) AND (fft[Filter]) AND (english[Filter])))) OR (Humans[Title/Abstract] AND ((fha[Filter]) AND (journalarticle[Filter] OR observationalstudy[Filter]) AND (fft[Filter]) AND (english[Filter]))) AND ((fha[Filter]) AND (journalarticle[Filter] OR observationalstudy[Filter]) AND (fft[Filter]) AND (english[Filter])))) AND ((((((((((((((Condom*[Title/Abstract] AND ((fha[Filter]) AND (journalarticle[Filter] OR observationalstudy[Filter]) AND (fft[Filter]) AND (english[Filter]))) OR ("Sexual Abstinence"[Title/Abstract] AND ((fha[Filter]) AND (journalarticle[Filter] OR observationalstudy[Filter]) AND (fft[Filter]) AND (english[Filter])))) OR (utilization*[Title/Abstract] AND ((fha[Filter]) AND (journalarticle[Filter] OR observationalstudy[Filter]) AND (fft[Filter]) AND (english[Filter])))) OR ("Unsafe Sex"[Title/Abstract] AND ((fha[Filter]) AND (journalarticle[Filter] OR observationalstudy[Filter]) AND (fft[Filter]) AND (english[Filter])))) OR ("Sexual Partners"[Title/Abstract] AND ((fha[Filter]) AND (journalarticle[Filter] OR observationalstudy[Filter]) AND (fft[Filter]) AND (english[Filter])))) OR (Practice*[Title/Abstract] AND ((fha[Filter]) AND (journalarticle[Filter] OR observationalstudy[Filter]) AND (fft[Filter]) AND (english[Filter])))) OR (prevention*[Title/Abstract] AND ((fha[Filter]) AND (journalarticle[Filter] OR observationalstudy[Filter]) AND (fft[Filter]) AND (english[Filter])))) OR (behaviors*[Title/Abstract] AND ((fha[Filter]) AND (journalarticle[Filter] OR observationalstudy[Filter]) AND (fft[Filter]) AND (english[Filter])))) OR (Prevalence*[Title/Abstract] AND ((fha[Filter]) AND (journalarticle[Filter] OR observationalstudy[Filter]) AND (fft[Filter]) AND (english[Filter])))) OR (Prevalence*[Title/Abstract] AND ((fha[Filter]) AND (journalarticle[Filter] OR observationalstudy[Filter]) AND (fft[Filter]) AND (english[Filter])))) OR ("Human papilloma virus"[Title/Abstract] AND ((fha[Filter]) AND (journalarticle[Filter] OR observationalstudy[Filter]) AND (fft[Filter]) AND (english[Filter])))) OR ("HIV Infections"[Title/Abstract] AND ((fha[Filter]) AND (journalarticle[Filter] OR observationalstudy[Filter]) AND (fft[Filter]) AND (english[Filter])))) OR ("Gonorrhea Infection"[Title/Abstract] AND ((fha[Filter]) AND (journalarticle[Filter] OR observationalstudy[Filter]) AND (fft[Filter]) AND (english[Filter])))) OR ("Sexually Transmitted infections"[Title/Abstract] AND ((fha[Filter]) AND (journalarticle[Filter] OR observationalstudy[Filter]) AND (fft[Filter]) AND (english[Filter]))) AND ((fha[Filter]) AND (journalarticle[Filter] OR observationalstudy[Filter]) AND (fft[Filter]) AND (english[Filter]))) |  | |
