# Supplementary material for: Preventive practices toward sexually transmitted infections and their determinants among young people in Ethiopia: A protocol for systematic review and meta-analysis
Source: PLoS One. 2022 Feb 3;17(2):e0262982. doi: 10.1371/journal.pone.0262982 (PMC8812866; doi:10.1371/journal.pone.0262982)
Supplement: S4 File — (DOCX) [file pone.0262982.s004.docx]

**Additional File 4**: JBI critical appraisals for observational studies as shown in the link below <https://jbi.global/critical-appraisal-tools>
