# Supplementary material for: Preventive practices toward sexually transmitted infections and their determinants among young people in Ethiopia: A protocol for systematic review and meta-analysis
Source: PLoS One. 2022 Feb 3;17(2):e0262982. doi: 10.1371/journal.pone.0262982 (PMC8812866; doi:10.1371/journal.pone.0262982)
Supplement: Attachment — Submitted filename: Response to Reviewers.docx [file pone.0262982.s005.docx]

Response to reviewer

#1

- Mother to fetus (line 48)
- Women and men … respectively (line 56-57.)
- STIs (line 76-77, 124, 126, 14-143, 182, 191, 195, 199, 201 )
- CINAHL (Line 129 )
- As searching in Google and Google scholar leads to numerous studies, limited number of studies will be screened from Google and Google scholar using this phrase “Preventive practices towards Sexually Transmitted Infection in Ethiopia” (line 130-132)
- Search strategy (additional file 2) (line 135)
- Filter will be applied on PubMed database on titles/abstract, full text, journal articles, observational studies, and English language to manage the available studies (additional file 2).
