# Supplementary material for: Preventive practices toward sexually transmitted infections and their determinants among young people in Ethiopia: A protocol for systematic review and meta-analysis
Source: PLoS One. 2022 Feb 3;17(2):e0262982. doi: 10.1371/journal.pone.0262982 (PMC8812866; doi:10.1371/journal.pone.0262982)
Supplement: Attachment — Submitted filename: Response to Reviewers.docx [file pone.0262982.s006.docx]

Response to reviewer

#1

- Scopus data base (line 28, 127 )
- Globally, the estimated annual new cases of curable sexually transmitted infections occurred among young people aged 15-24 years was about 178.5 million ((line 20-21 ). 178.5 million new cases was just to estimate about half of the 357 million cases among people aged 15–49 years were between 15-24 years. i.e 50% of 357 ≈178.5 (line 55)
- Globally, the estimated annual new cases of curable sexually transmitted infections occurred among people aged 15–49 years was 357 million, and about half of them were between 15-24 years (line 54-55).
- CINAHL (line 27)
- STIs (line 45, 54, 56, 64, 74, 86,88, )
- human immunodeficiency virus (HIV) (line 48)
- STIs cause (line 59)
- contraceptive methods (line 67)
- STIs remain (line 73)
- Both quantitative and qualitative data (line 143)
- National survey. It was mistake and corrected now (line 145)
